# Evidence for a Transport-Trap Mode of *Drosophila melanogaster gurken* mRNA Localization

**DOI:** 10.1371/journal.pone.0015448

**Published:** 2010-11-12

**Authors:** Lan Lan, Shengyin Lin, Sui Zhang, Robert S. Cohen

**Affiliations:** Department of Molecular Biosciences, University of Kansas, Lawrence, Kansas, United States of America; Columbia University, United States of America

## Abstract

The *Drosophila melanogaster gurken* gene encodes a TGF alpha-like signaling molecule that is secreted from the oocyte during two distinct stages of oogenesis to define the coordinate axes of the follicle cell epithelium that surrounds the oocyte and its 15 anterior nurse cells. Because the *gurken* receptor is expressed throughout the epithelium, axial patterning requires region-specific secretion of Gurken protein, which in turn requires subcellular localization of *gurken* transcripts. The first stage of Gurken signaling induces anteroposterior pattern in the epithelium and requires the transport of *gurken* transcripts from nurse cells into the oocyte. The second stage of Gurken signaling induces dorsovental polarity in the epithelium and requires localization of *gurken* transcripts to the oocyte's anterodorsal corner. Previous studies, relying predominantly on real-time imaging of injected transcripts, indicated that anterodorsal localization involves transport of *gurken* transcripts to the oocyte's anterior cortex followed by transport to the anterodorsal corner, and anchoring. Such studies further indicated that a single RNA sequence element, the GLS, mediates both transport steps by facilitating association of *gurken* transcripts with a cytoplasmic dynein motor complex. Finally, it was proposed that the GLS somehow steers the motor complex toward that subset of microtubules that are nucleated around the oocyte nucleus, permitting directed transport to the anterodorsal corner. Here, we re-investigate the role of the GLS using a transgenic fly assay system that includes use of the endogenous *gurken* promoter and biological rescue as well as RNA localization assays. In contrast to previous reports, our studies indicate that the GLS is sufficient for anterior localization only. Our data support a model in which anterodorsal localization is brought about by repeated rounds of anterior transport, accompanied by specific trapping at the anterodorsal cortex. Our data further indicate that trapping at the anterodorsal corner requires at least one as-yet-unidentified *gurken* RLE.

## Introduction

The localization of mRNAs to specific subcellular sites is a common mechanism by which cells target proteins to regions where they are needed and/or prevent them from accumulating in places where they may do harm. While localized mRNAs have been described in all examined organisms, genome-wide analyses have been limited to Drosophila [1], where it has been estimated that 71% of all transcripts are localized. Localized mRNAs encode a variety of proteins types including components of the cytoskeleton, transcription factors, regulators of translation, and even secreted signaling molecules [1].

Three distinct mechanisms have been described for mRNA localization. These include directed transport on microtubule (MT) or, more rarely, actin tracks, diffusion to a localized anchor, and region-specific mRNA degradation [2]–[6]. All three mechanisms are mediated by discrete RNA localization elements (RLEs) that recruit localization machineries to their respective transcripts through specific RNA-protein interactions. The vast majority of characterized RLEs reside in the 5′ or 3′ untranslated regions (UTRs) of their transcripts, although a few have been mapped to protein coding regions [5]. A fourth mechanism of mRNA localization, transcription from a subset of syncytial nuclei, is transcription-based and does not require RLEs *per se* [6]–[8].

One of the best systems for studying mechanisms of mRNA localization is the Drosophila oocyte whose maturation and patterning is dependent on a cascade of mRNA localization events [9]. The oocyte develops within an egg chamber composed of an outer, somatically-derived follicle cell epithelium and an inner germ-line cyst that includes a single posterior oocyte and 15 sister nurse cells [9]. The vast majority of mRNAs found in the developing oocyte, mature egg, and syncytial embryo are synthesized in nurse cells during early stages of oogenesis (i.e., stages 1–6) and transported into the oocyte through cytoplasmic bridges, remnants of incomplete cytokinesis during germ-line cyst formation [3]. Such transport is powered by cytoplasmic dynein [10], [11], a minus end-directed MT motor protein, and initially results in the accumulation of the transported transcripts at the oocyte's posterior pole, which contains a prominent MT organizing center (MTOC) [2], [12]. Due to their continued association with cytoplasmic dynein and programmed reorganization of the oocyte's MT cytoskeleton, all transported RNAs (including *gurken*, see below) are relocalized to the oocyte's anterior cortex at stage 7 and form a characteristic ring-like distribution pattern [2], [10], [11], [13].

Most transported mRNAs persist at the anterior cortex through stage 10, when a final MT reorganization event induces vigorous cytoplasmic streaming [14] that causes the RNAs to become dispersed throughout the ooplasm. *bicoid* mRNA, which encodes a transcription factor morphogen that patterns the anterior end of the future embryo, is an exception. It becomes anchored to the actin cortex and thus remains localized through cytoplasmic streaming and into early embryogenesis [2], [9], [15]. Other transported RNAs remain at the anterior cortex only transiently and are instead relocalized to other sites. These include *oskar* and *nanos*, which are both relocalized to the posterior pole and encode proteins that pattern the posterior portion of the future embryo [2]. In the case of *oskar*, such relocalization occurs during stage 8/9 and involves association of the mRNA with a plus end motor complex that includes Kinesin I [4], [10], [16]–[18]. In the case of *nanos* mRNA, such relocalization is delayed until stage 10 and is mediated by a diffusion trap mechanism in which cytoplasmic streaming and Oskar protein facilitate diffusion and trapping, respectively [19].

*gurken* and transcripts encoded by the *I Factor* retro-transposon [20] are the only known transported RNAs that are relocalized to the oocyte's anterodorsal corner. Such relocalization begins during stage 8 (i.e., shortly after anterior localization) and persists through stage 10 [21]–[23]. Previous studies have reported that an RNA element within the *gurken* protein coding region, called the GLS (*gurken* localization sequence), is both required and sufficient for transient (e.g., stage 8/9 only) localization of injected *gurken* transcripts to the AD corner of the oocyte [20]. Such localization was described to be MT- and cytoplasmic dynein-dependent and to involve directed transported from the anterior cortex to the AD corner. From these data, it was proposed that MTs nucleated around the oocyte nucleus are somehow different than those nucleated at other regions of the anterior cortex and that the GLS “steers” *gurken* mRNA-cytoplasmic dynein motor complexes toward the former. Here we re-investigate the role of the GLS in *gurken* mRNA localization using a transgenic fly assay system that includes use of the endogenous *gurken* promotor and both biological rescue and RNA localization assays of GLS activity. In contrast to the previous studies [23], we find that the GLS is sufficient for transport into the oocyte and anterior localization, but not for anterodorsal localization, transient or otherwise. Our data are consistent with a model in which AD localization is brought about by repeated rounds of transport to the anterior cortex, coupled with specific anchoring of the transcripts around the oocyte nucleus in the AD corner of the cell. Presumably, such anchoring is mediated by RLEs other than the GLS, although we cannot rule out the possibility the GLS functions with such other RLEs to facilitate anchoring.

## Results

### Identification of a highly conserved sequence element with predicted stem-loop secondary structure in the *gurken* protein coding region

**Figure 1:**
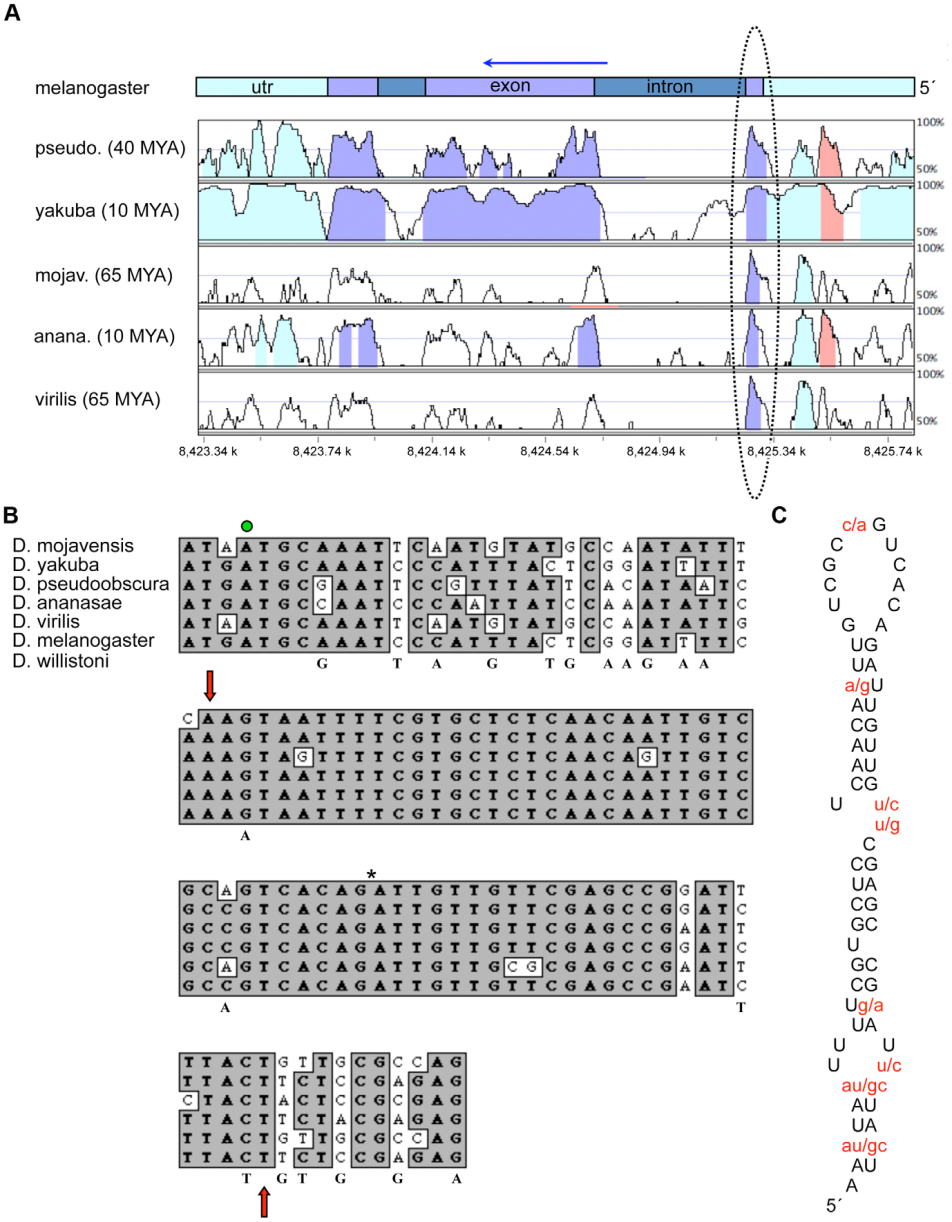
Conservation and predicted secondary structure of the GLS. (A) Sequence alignment of the *gurken* transcription unit displayed using the Vista Browser at http://pipeline.lbl.gov/cgi-bin/gateway2 [49]. The estimated years in millions (MYA) of evolution between *D*. *melanogaster* and each of the other five species is from Heger and Ponting [24]. The most highly conserved region is circled and includes the first 39 nt of the GLS. The last 25 nt of the GLS map to the 3′ side of the abutting intron. The arrow indicates the direction of transcription. The red shaded region corresponds to a putative transposable element. The numbers at the bottom of the graph indicate nucleotide position along the chromosome. (B) The 5′ end of the *gurken* mRNA, where the green dot denotes the translation start site, the red arrows the boundaries of the GLS, and the asterisk the position of the intron. The nucleotides beneath the aligned sequence blocks highlight differences between the *D. Willistoni* and *D. melanogaster* sequences. (C) Predicted secondary structure of the GLS, with non-conserved residues shown in red.

It was clear from our previous attempts to map *gurken*'s RLEs that one or more such elements are located in the protein coding portion of the gene [21]. While the vast majority of known RLEs do not exhibit strong sequence conservation across species, we reasoned that an RLE in the *gurken* protein coding sequence might since it would be under dual selective pressure, one to maintain a functional protein and another to maintain recognition by the RNA localization machinery. We thus aligned *gurken* gene sequences from six different Drosophila species, separated by 10 to 65 million years of evolution, and looked for 40 nucleotides (nt) or longer sequence elements in the protein coding portion of the gene that were at least 90% identical. As seen in Figs. 1A and B, a single such element was identified. Database searches indicated that the identified sequence, which corresponds to amino acid residues 10–31, is the same as the GLS reported by Van de Bor et al. [20]. As previously recognized by them, the conservation of the GLS among different Drosophila species is even more striking at the level of predicted secondary structure. Indeed, all six GLSs are predicted to form the exact same stem-loop secondary structure (Fig. 1C) and to encode the exact same protein sequence. To address the possibility that the highly conserved nature of the GLS is somehow reflective of codon preference (rather than maintenance of a particular secondary structure), we also examined the GLS of *D. willistoni*, which has a different codon preference than the six species used in our initial alignment [24]. We found that the *D. willistoni* GLS only differs from the *D. melanogaster* GLS at four nucleotide residues and encodes the exact same predicted secondary structure (Fig. 1C). It is also noteworthy, that the codons outside of the GLS vary from species to species, which would not be expected if codon choice was under high selective pressure, e.g., as a means to tightly control Grk protein levels.

### The GLS possesses anterior, but not AD, localization activity

**Figure 2:**
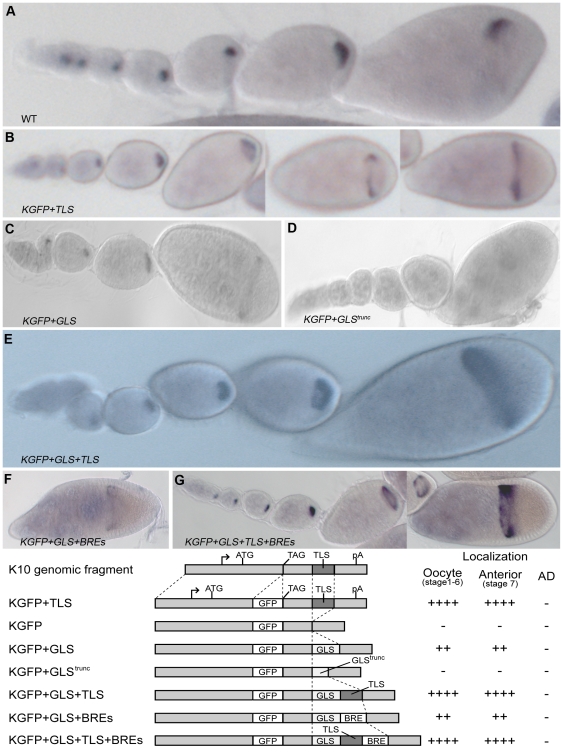
The GLS is sufficient for anterior, but not anterodorsal localization within the Drosophila oocyte. RNA distribution patterns of wild-type *gurken* transcripts (A) and *K10::GFP* reporter transcripts (B–G) as revealed by wholemount *in situ* hybridization (see Methods). Individual ovarioles are shown, with older egg chambers oriented to the right. The transgenes (B–G) are noted in the individual panels. The structure of the transgenes and expression summary is shown beneath the *in situ*s.

**Figure 3:**
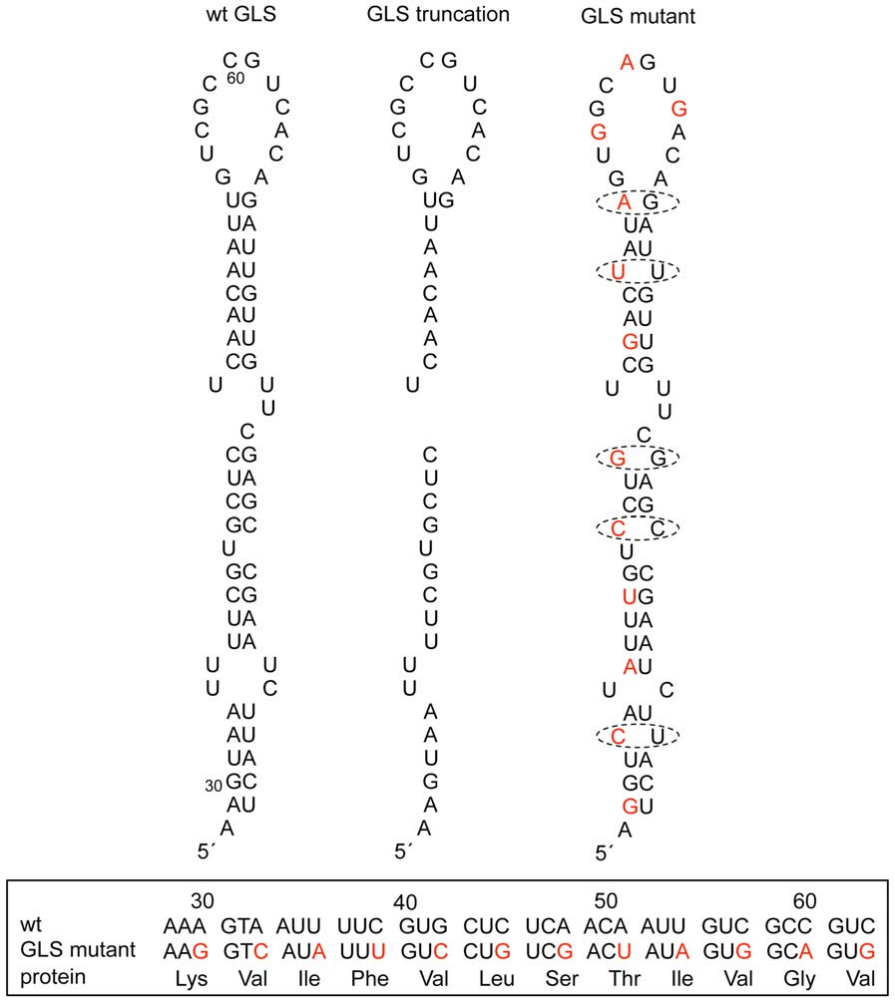
Structure of GLS variants. The wild-type GLS is shown at the left for comparison. The GLS mutant (referred to as *grkGLS^mut^* in Text) contains 12 point mutations (shown in red), which are predicted to disrupt the predicted base pairing pattern of the GLS at five sites (circled). None of the 12 mutations affect the protein coding sequence as shown at the bottom portion of the figure.

To determine if the GLS possesses RNA localization activity, we used a transgenic fly assay system. The starting point for these studies was a *K10::GFP* reporter gene construct (called *KGFP*, Fig. 2) that contains the *K10* nurse cell enhancer/promoter region and the bulk of the *K10* transcription unit, including the poly(A) addition signal, but lacks the *K10* RLE (called the TLS) [25]. As expected, the *KGFP* transgene produced transcripts that exhibited no localization activity (data not shown), i.e., they remained in nurse cells until very late stages (i.e., after stage 11) of oogenesis, when nurse cells indiscriminately dump their entire cytoplasmic contents into the oocyte, in a process known as nurse cell regression. We next inserted a wild-type or truncated copy of the GLS (Fig. 3) into the 3′UTR of the *KGFP* reporter and introduced the resulting constructs, called *KGFP+GLS* and *KGFP+GLS^trunc^*, respectively into flies. We found that *KGFP+GLS* transcripts accumulated in the oocyte beginning at about stage 2 and steadily increased in abundance through about stage 6 or 7, when they became localized to the oocyte's anterior cortex and formed the same ring-like distribution pattern observed for endogenous *gurken* and other transported transcripts (Fig. 2C). However, in contrast to wild-type *gurken* transcripts (Fig. 2A), the anterior ring of *KGFP+GLS* transcripts persisted through stage 10, and did not refine itself into the AD cap in any of more than 200 examined stages 9 and 10 egg chambers from each of 4 different transgenic lines. As expected, *KGFP+GLS^trunc^* transcripts exhibited no localization activity (Fig. 2D). We tentatively conclude from these findings that the GLS possesses anterior, but not AD, localization activity.

The transport of *KGFP+GLS* transcripts into the oocyte is less robust than the transport of endogenous *gurken* transcripts or the transcripts of *KGFP* transcripts that contain the *K10* TLS (compare Fig. 2C to 2A and 2B). This led us to wonder if the inability of the GLS to mediate AD localization was due to its inability to bind the transport machinery tightly. To address this concern, we inserted a copy of the *K10* TLS into the *KGFP+GLS* reporter construct to make *KGFP+GLS+TLS*. As seen in Fig 2E, *KGFP+GLS+TLS* transcripts exhibited robust transport into the oocyte and strong anterior localization, indicating that they bind the transport machinery tightly. However, *KGFP+GLS+TLS* transcripts never became enriched at the AD corner of the cell, supporting our earlier conclusion that the GLS lacks AD localization activity.

We next wondered whether the inability of the GLS to mediate AD localization was related to the fact that *KGFP* transcripts, with or without the GLS and/or TLS, are translated. Endogenous *gurken* transcripts are translationally repressed during their relocalization from the anterior cortex to the AD corner [26] and we were concerned that such repression is necessary for relocalization. Consistent with this idea, recent studies have shown that wild-type *gurken* transcripts are highly dynamic, except at sites of translation activation, i.e., the oocyte's AD corner [27]. The repression of *gurken* translation is thought to be brought about by the binding of a protein complex consisting of Cup, Squid, PABP55B and Bruno to Bruno Response Elements (BREs) located in the 3′UTR of *gurken* mRNA [26]. Consistent with this idea, in vivo *gurken* signaling activity is highly responsive to alterations in Bruno expression levels [28], [29]. To determine whether BRE elements confer AD localization activity onto the GLS element, we inserted the same three copies of the BRE from the *oskar* 3′ UTR that faithfully repress *oskar* translation [28], [30] into the *KGFP+GLS* and *KGFP+GLS+TLS* reporter constructs. As seen in Figs. 2F and G, the BREs did not alter GLS localization activity, i.e., *KGFP+GLS+BREs* and *KGFP+GLS+TLS+BREs* transcripts were localized to the anterior cortex normally, but never relocalized to the AD corner We also saw no AD localization when *KGFP* transcript distribution patterns were assessed by confocal microscopy and fluorescence probes (data not shown) rather than by the enzyme linked detection scheme used in Fig. 2. We conclude from these findings that the GLS is unable to mediate AD localization even in the presence of BRE elements. The one caveat to these experiments is that the BRE elements failed to noticeably repress the translation of *K10::GFP* transcripts; we detected similar amounts of GFP fluorescence in the nuclei of flies carrying transgenes with BRE elements as with flies carrying transgenes without BRE elements. Why the BRE elements failed to repress the translation of the *K10::GFP* transcripts is not clear, but these findings suggest that the transport complexes assembled by the GLS alone are somehow different than the ones assembled by intact *gurken* transcripts and that these differences are critical for BRE-mediated translation repression.

While the simplest interpretation of above findings is that the GLS lacks AD localization activity, we cannot rule out the possibility that GLS possesses AD localization activity but that such activity is somehow masked by flanking sequences in the *K10::GFP* reporter transcript. We think this is unlikely for a couple of reasons, however. First, the GLS was inserted into the same general region of the reporter transcript that supports TLS RNA activity, which like that of the GLS appears to rely on the formation of a simple stem-loop secondary structure [24]. Second, the first 25–50 nt that flank the GLS in the *KGFP+GLS, KGP+GLS+TLS, KGP+KGFP+GLS+BREs* and *KGFP+GLS+TLS+BREs* transcripts all differ from one another, yet GLS localization activity remains constant. Another possibility is that AD localization activity is somehow lost when the GLS is moved from a protein coding to a non-protein coding portion of the transcript. While we have not tested this possibility, it is noteworthy that previous studies have indicated that the GLS “retains” AD localization, even when located downstream of the protein coding portion of a GFP reporter transcript [20].

### The generation of a *gurken* RNA null allele

**Figure 4:**
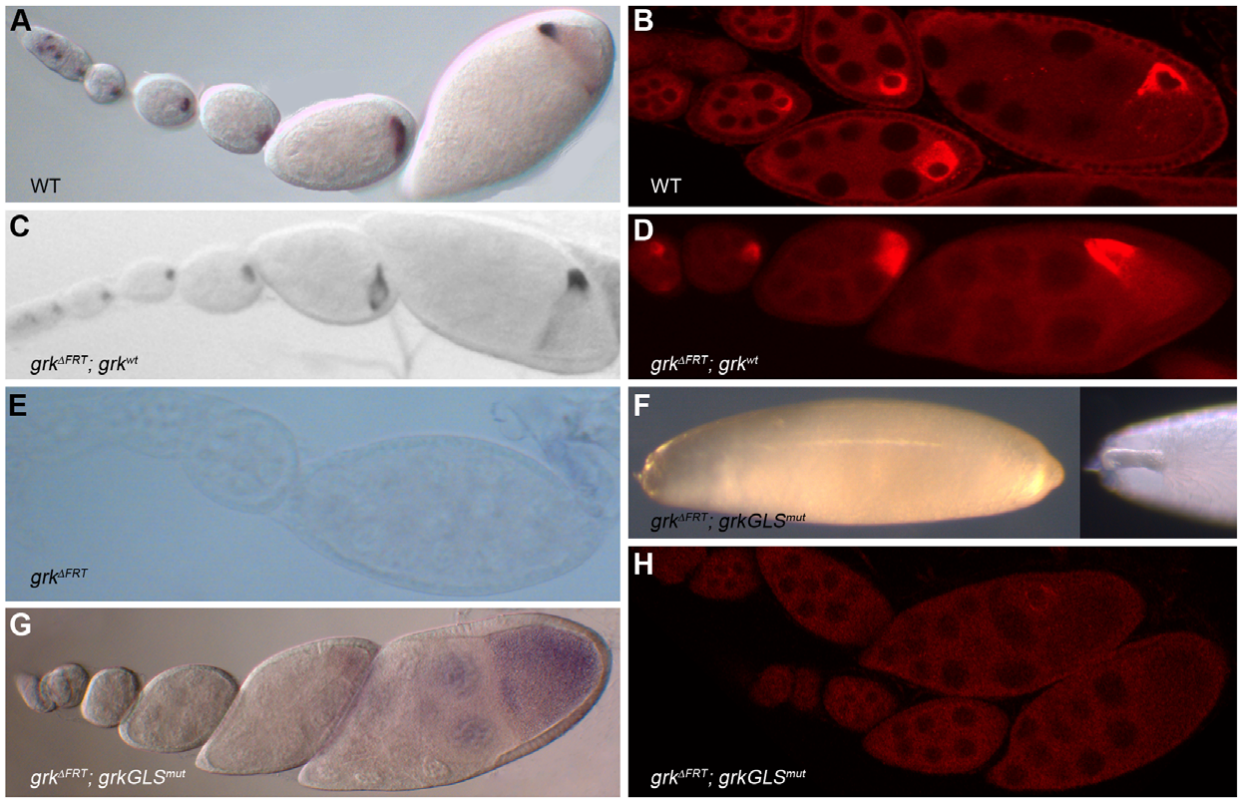
The GLS is required for *gurken* RNA localization and gene function. (A–B) Wild-type expression patterns of endogenous *gurken* RNA (A) and protein (B) as revealed by whole mount *in situ* hybridization and immunofluorescence, respectively. Anterodorsal localization of transcripts and protein is only apparent in the rightmost egg chambers, which are stage 8 and 9, respectively. (C–E) The *gurken* RNA and protein distribution patterns of *gurken* null mutants (*grk^ΔFRT^*) carrying the wild-type *gurken* transgene, *grk^wt^* (C–D) or no transgene (E). (F–H) *grk^ΔFRT^* eggs and egg chambers (from *gurken* null mothers) carrying the *grkGLS^mut^* transgene. (F) Left panel: representative *grk^ΔFRT^*; *grkGLS^mut^* egg exhibiting a completely ventralized phenotype, i.e., complete loss of dorsal appendage material. Right panel; anterior end of a *grk^ΔFRT^*; *grkGLS^mut^* egg exhibiting a strong, but not complete, ventralized phenotype. Note, for example the short, fused dorsal appendage. (G) *grk^ΔFRT^*; *grkGLS^mut^* ovariole following *in situ* hybridization with *gurken* probe. Transcripts are dispersed throughout the germ-line cysts with only slight enrichment in the oocyte and no subcellular localization. (H) *grk^ΔFRT^*; *grkGLS^mut^* ovariole following immunofluorescence using an anti-Grk antibody. The protein is generally dispersed throughout the germ-line cysts, although slight enrichment around the oocyte nucleus is seen in rare stage 10 and 11 egg chambers.

We next wanted to study the role of the GLS in *gurken* RNA localization and gene function within the context of a more wild-type transcript. To this end, we first set out to generate a *gurken* RNA null allele, so that we could detect *gurken* transgene transcripts without having to mark them with heterologous sequence tags, which could compromise *gurken* gene function. Previously described *gurken* null alleles are not complete deletions and produce significant amounts of *gurken* transcripts (unpublished observations). We were fortunate that the Exelixis stock collection includes lines that carry FRT elements just outside the 5′ and 3′ ends of the *gurken* transcription unit (see Methods). We successfully used these lines along with one that carries the FLP recombinase to generate a complete deletion allele of the *gurken* gene, called *grk^ΔFRT^*. Homozygous *grk^ΔFRT^* flies are viable, but the females are completely sterile and produce egg chambers with no detectable *gurken* transcripts (Fig. 4E).

The egg chambers and eggs produced by homozygous *grk^ΔFRT^* flies exhibited severe anteroposterior and dorsoventral patterning defects, consistent with previous studies which have identified two distinct functions for *gurken* during Drosophila oogenesis [31]–[34]. The first of these functions is the induction of anteroposterior asymmetry in the follicle cell epithelium that surrounds the nurse cell-oocyte cluster. Following the transport of *gurken* mRNA into the oocyte and translation, Gurken protein (Grk) is secreted locally and induces neighboring follicle cells to adopt the posterior cell fate [31]–[33]. At stage 7 of oogenesis, posterior follicle cells send a signal back to the oocyte that polarizes the oocyte's MT cytoskeleton, a prerequisite both for the migration of the oocyte nucleus to a point along the oocyte's anterior cortex [31], and the transport of *bicoid*, *oskar* and other mRNA that encode embryonic patterning determinants to specific ends of the oocyte [2], [4]. *gurken'*s second function is that of inducing dorsoventral asymmetry in the follicle cell epithelium. Following the relocation of its mRNA to the oocyte's anterodorsal corner and translation [22], [23], Grk is secreted locally and induces neighboring follicle cells to adopt the dorsal cell fate [35], [36]. Dorsal and ventral (non-induced) follicle cells subsequently differentially signal the oocyte, polarizing the dorsoventral axes of the mature egg and future embryo.

Similar to analyses of other *gurken* null and strong loss-of-function alleles [37], we find that *grk^ΔFRT^* females lay very few eggs and those that are laid are completely ventralized, most readily evident by their elongated shape and absence of dorsal appendages on their eggshells (data not shown). Such eggs are also translucent and extremely fragile, suggestive of a general defect in follicle cell differentiation and/or cell fate determination. Also as expected, *grk^ΔFRT^* females exhibited strong defects in the specification of anteroposterior polarity as evident by their inability to support nuclear migration and/or the localization of *K10* transcripts to the anterior cortex of stage 7 oocytes (data not shown). We also found that *grk^ΔFRT^* females produce a high percentage (several per ovariole) of compound egg chambers, i.e., egg chambers that contain two or more germ-line cysts encased in a single follicle cell epithelium. All of these phenotypes—female sterility, ventralized and fragile eggs, and compound egg chambers—are due to the loss of *gurken* gene function, since they were completely rescued by the introduction of a wild-type *gurken* transgene, *grk^wt^* into the germ-line (Fig. 4C, and see Methods).

### The GLS is required for *gurken* gene function

To determine if the GLS is required for normal *gurken* gene function, we created a rescue construct, called *grkGLS^mut^*, that is identical to *grk^wt^*, except for the inclusion of 12 single-base mutations, all within the GLS. All 12 mutations target wobble nucleotides and preserve the encoded protein sequence (Fig. 3). Five of the mutations disrupt the predicted base pairing pattern of the GLS (Fig. 3) and according to mFOLD (http://mfold.bioinfo.rpi.edu/) are sufficient to destabilize the wild-type structure (data not shown). The other seven mutations disrupt the primary sequence only, but such mutations in other RLEs (e. g., see [38]) are known to compromise RNA localization activities. Four independent lines carrying the *grkGLS^mut^* transgene were crossed into a *grk^ΔFRT^* background for analysis. RT-PCR analyses revealed significant (∼10-fold) variation in the level of transgene transcript accumulation. Two of the lines exhibited wild-type or near wild-type levels of accumulation and we focused on them for all subsequent analyses. Such analyses revealed no significant differences in the behavior or activities of these two transgenic lines and thus we describe them below as if they are a single line/transgene.

The *grkGLS^mut^* transgene exhibited no or only weak rescue of the dorsoventral patterning defects of *grk^ΔFRT^* flies; ∼50% of the recovered eggs (n>1000 per line) were fully ventralized and similar to those produced by *grk^ΔFRT^* flies. The remaining recovered eggs were strongly ventralized, containing a single small appendage on the dorsal midline (Fig. 4F). The *grkGLS^mut^* transgene exhibited much better, but still not complete rescue of the anteroposterior patterning defects of *grk^ΔFRT^* flies. Thus while nuclear migration was consistently delayed and sometimes (5–25% of the time) incomplete, most stage 8 and older egg chambers contained a correctly positioned nucleus and exhibited wild-type localization of *K10* mRNA to the anterior cortex (data not shown). We conclude from these data that the GLS is required for *gurken'*s anteroposterior and, especially, dorsoventral patterning activities, both of which are dependent on faithful transport and subcellular localization of *gurken* transcripts. The *grkGLS^mut^* transgene rescued all other *gurken* activities; *grk^ΔFRT^; grkGLS^mut^* flies produced virtually no compound egg chambers (only two compound egg chambers were detected in more than 50 examined ovaries or about ∼1000 egg chambers from each of the two extensively examined lines), and none of the recovered eggs (n>1000 per line) were fragile. We also saw a general increase in viability of *grk^ΔFRT^; grkGLS^mut^* flies compared to *grk^ΔFRT^* controls; we recovered many more non-CyO flies from sibling crosses of *grk^ΔFRT^; grkGLS^mut^*/CyO flies than from sibling crosses of *grk^ΔFRTt^*/CyO flies. We interpret such rescue to mean that mutations in the GLS do not interfere with *gurken* transcription or translation, but rather only mRNA localization.

### The GLS is required for the localization of *gurken* transcripts

Given the moderate rescue of the anteroposterior patterning defects of *grk^ΔFRT^* ovaries by the *grkGLS^mut^* transgene, we expected only modest defects in the transport and anterior localization of *grkGLS^mut^* transcripts. Unexpectedly, *in situ* hybridization experiments revealed no enrichment of *gurken* transcripts in stage 1–7 oocytes of *grkGLS^mut^*; *grk^ΔFRT^* flies, and the transcripts never became concentrated along the oocyte's anterior cortex (Fig. 4G). Significantly more *grkGLS^mut^* transcripts were detected in later stage (e.g., stage 8–10) oocytes, but this is likely due to diffusion, since the diameter of the cytoplasmic bridges between nurse cells and the oocyte increases dramatically during these stages (unpublished observations). We conclude from these findings that the GLS is required for the transport and anterior localization of *gurken* transcripts, and strongly suspect that this requirement is met by the GLS's ability to recruit a cytoplasmic dynein motor complex. Whether the small amounts of *gurken* transcripts detected in stage 1–7 *grkGLS^mut^*; *grk^ΔFRT^* oocytes is indicative of residual GLS transport activity, the transport activity of other RLEs in the *gurken* mRNA, and/or diffusion of *gurken* transcripts from nurse cells into the oocyte is not clear from our data, although the complete absence of anterior localization is most supportive of the last possibility. Antibody stains for Grk protein were consistent with the RNA data. Most stage 1–7 egg chambers showed no obvious enrichment of Grk protein in the oocyte or anywhere else in the germ line cyst (Fig. 4H). Given that most *grkGLS^mut^*; *grk^ΔFRT^* oocytes supported nuclear migration and *K10* mRNA localization, we further conclude from these analyses that very low levels of *gurken* mRNA and protein are sufficient for anteroposterior patterning and that such patterning does not require subcellular localization of *gurken* transcripts and/or protein within the oocyte.

The rescue data predicts a stronger requirement for the GLS in the localization of transcripts to the oocyte's AD corner. Thus while the *grkGLS^mut^* transgene showed moderate rescue of the anteroposterior defects of *grk^ΔFRT^* egg chambers and eggs, it exhibited almost no rescue of the dorsoventral patterning defects of *grk^ΔFRT^* ovaries (see above). Consistent with this prediction, the *grkGLS^mut^* transcripts were dispersed throughout the ooplasm in all examined staged 7–10 *grkGLS^mut^*; *grk^ΔFRT^* oocytes. Antibody stains for Grk protein were again consistent with the RNA data in that the vast majority of examined stage 8–10 egg oocytes showed no enrichment of Grk protein around the nucleus or elsewhere in the cell. Surprisingly, however, a few (less than 5%) showed distinct enrichment of Grk protein around the oocyte nucleus (e.g., see Fig. 4H). We interpret such enrichment to mean that *gurken* translational activator and/or derepressor proteins are concentrated around the oocyte nucleus, which could also explain the residual dorsoventral patterning activity of the *grkGLS^mut^* transgene despite its absence of AD RNA localization activity.

## Discussion

The major finding of our studies is that the GLS is required but not sufficient for AD localization. We interpret this to mean that AD localization is brought about by the action of the GLS plus one or more additional RLEs, henceforth referred to as AD-RLEs. The nature of the requirement for the GLS in AD localization is not clear, but may simply be that of getting *gurken* transcripts to the oocyte's anterior cortex, where they can associate (through the action of the AD-RLEs) with the AD localization machinery. Consistent with this view, transgenic RNAs that contain the GLS but no other *gurken* RLEs, e.g., *KGFP+GLS* transcripts (Fig. 2), are transported into the oocyte and subsequently accumulate along the oocyte's anterior cortex, but never become concentrated at the oocyte's AD corner. The transport and anterior localization pattern of *KGFP+GLS* transcripts is mirrored by a number of other mRNAs, including *K10* and *Orb* and is completely consistent with the idea that the GLS mediates association of *gurken* mRNA with a minus end motor complex, most probably cytoplasmic dynein. Direct support for this idea comes from real-time imaging and immunoelectron microscopy experiments [39], which show that *gurken* transcripts form large particles that contain cytoplasmic dynein heavy chain (DHC) and the motor cofactors Egalitarian (Egl) and Bicaudal D (BicD) upon injection into stage 7–9 oocytes. Moreover, the majority of these particles are in close proximity to microtubules and their formation is dependent on the GLS. Finally, it has been shown that Dynein light chain (Ddlc) binds *gurken* mRNA *in vitro* and that such binding is mediated by the 3′UTR, not the GLS [40].

How the AD-RLEs mediate the relocalization of *gurken* transcripts from the anterior cortex to the AD corner is not clear, although one simple possibility is that they bind proteins that are (or become) anchored to the oocyte nucleus or to a neighboring structure. Given that some *grkGLS^mut^* transcripts accumulate in (diffuse into?) the oocyte, yet only very inefficiently become concentrated at the AD corner (or around the nucleus), it would appear that efficient AD localization requires active transport, i.e., AD localization cannot be brought about by diffusion within the oocyte and specific anchoring at the AD corner of the cell. However, it is not clear as previously suggested [20] that such transport needs to be specifically directed toward the AD corner. Rather AD localization could be brought about by repeated rounds of GLS-mediated transport to the anterior cortex (i. e., to the minus ends of the oocyte's MTs), coupled with region-specific anchoring at the AD corner and/or to the nucleus. In this scenario, the AD-RLEs, which could include the GLS, would constitute the RNA component of the anchor complex. Consistent with the notion of anchoring, photo-bleaching and real-time imaging experiments reveal that endogenous and injected *gurken* transcripts are highly dynamic during early and middle stages of oogenesis, but become static coincident with AD localization [27], [39]. The dynamic to static transition is accompanied by a distinct change in *gurken* particle morphology [39]. Interestingly, this transition requires cytoplasmic dynein, but not other components of the motor complex, e.g., Egl and BicD. These observations have led to the proposal that upon reaching its final destination, the Dynein motor becomes a static anchor and is no longer a functional motor protein.

How do we reconcile our findings with those of previous studies [20] which indicate that the GLS mediates directed transport to the oocyte's AD corner? One possibility relates to the fact that such studies utilized either injected RNAs or transgenic RNAs expressed from very strong promoters. Both scenarios are likely to result in the formation of very large transport particles and it may be that such particles are better able to recruit the AD localization machinery than endogenous *gurken* transport particles, e.g., because of a higher number of GLS elements within the partcile. It should also be noted that the AD localization activity of injected GLS-containing transcripts is not nearly as complete as the AD localization of wild-type *gurken* transcripts; wild-type *gurken* transcripts are rarely detected outside the AD corner of stage 9 oocytes, whereas injected transcripts are readily detectable in all regions of the anterior cortex [20]. Similalry, the AD localization of previously described GLS-containing transgene transcripts is transient in nature, not persisting past stage 9. Taken together, these data indicate that the GLS is not sufficient (even in multiple copies) for wild-type AD localization, and that one or more additional elements are needed for persistent AD localization

It is not clear how cytoplasmic dynein switches from a motor to anchor. Thus while *gurken* transport and anchor particles are morphologically distinct, no proteins have been identified that are specific to one particle or the other. Given that *gurken* transcripts are specifically translated at the AD corner of the oocyte, the switch might be regulated by translation. Consistent with this idea, *gurken* transcripts never become anchored (remain dynamic) in *K10* and *Squid* mutants and are translated all along the anterior cortex [27]. Squid is a normal component of *gurken* transport and anchor particles, but is specifically required for anchoring. Thus, the switch from transport to anchoring might involve some sort of activation of Squid. K10 is an attractive candidate here as it binds Squid in vitro [41]. Moreover, K10 is concentrated in the oocyte nucleus and thus could provide the necessary asymmetry to the system. The one obvious caveat to this scenario is that K10 appears to be strictly nuclear and tightly associated with the oocyte's chromatin. Squid, while predominantly a cytoplasmic protein, is also detected in the nucleus and has been shown to interact with transportin, a nuclear import protein in vitro [41]. The activation of Squid and *gurken* anchoring could thus be brought about by specific modification of Squid in the oocyte nucleus by K10.

## Methods

### Drosophila genetics

Fly culture and crosses were carried out according to standard procedures [42]. The wild-type stock was *w^1118^*. The *gurken* deletion (*grk^ΔFRT^*) was made by inducing recombination [43] between the FRT insertions (FRT9855 and FRT7069, respectively) of stocks f07069 and d09855 (Harvard Medical School Exelixis collection). The resulting deletion, which extends from 73 nt upstream of the *gurken* transcription start site to ∼1100 nt downstream of the *gurken* poly(A) addition site unit was initially identified by non-complementation with *grk^2E^* [37] and subsequently confirmed by PCR analysis. Homozygous *grk^ΔFRT^* flies are viable, but female sterile (see Text), and maintained over the CyO chromosome balancer. The female sterility is completely rescued by introduction of a genomic copy of the wild-type *gurken* gene (see text). Homozygous *grk^ΔFRT^* females were identified by their straight (Cy^+^) wings. A complete description of all alleles and balancer chromosomes is found at http://flybase.bio.indiana.edu.

### P element transformations and transgene constructs

All constructs were cloned into the pCaSpeR4 vector [44] for introduction into the Drosophila germ-line. P-element mediated transformation of *w^1118^* flies was carried out as previously described [21], [25]. At least two lines were generated and analyzed for each construct. Most of the transgene lines were maintained as homozygous stocks. Transgene insertions that were homozygous lethal were maintained over the *TM3, Sb* balancer chromosome.

*The* K10::GFP *fusion constructs:* The starting point for these constructs was a 3.1 kb fully functional *K10* genomic clone that extends from a natural *Asp718 I* restriction site ∼850 nt upstream of the transcription start site to a natural *Sal I* site ∼400 nt downstream of the poly(A) addition site [13]. We then used PCR technology to remove the *K10* translation stop site and insert a 750 nt GFP fragment in-frame with the *K10* protein coding region. Finally, we replaced an ∼300 nt *Stu I – Hpa I* restriction fragment in the *K10* 3′UTR that includes the TLS RLE, with a *Bgl II – Xho I* linker. The resulting construct, called *KGFP*, produces readily detectable amounts of RNA and protein that are retained in nurse cells until nurse cell regression at stage 11 (data not shown). All *KGFP* variant constructs (see Fig. 2 and Results) were made by inserting appropriate synthetic linker DNAs (sequences available upon request) into the *Bgl II – Xho I* sites of *KGFP* and cloned into the *Asp718 I – Xba I* sites of the pCaSpeR4 vector for P element-mediated transformation.

*The* grk^wt^ *and* grkGLS^mut^ *rescue constructs:* The starting point for these constructs was a 14.1 kb *gurken* genomic fragment that extends from a natural *Asp718 I* site ∼7.1 kb upstream of the transcription start site to a synthetic *Spe I* site ∼300 nt downstream of the poly(A) addition site. To make *grk^wt^*, the 14.1 kb genomic fragment was cloned directly into the *Asp718 I – Xba I* sites of the pCaSpeR4 transformation vector. To make *grkGLS^mut^*, the GLS-containing *Sap I – Hind III* region of *grk^wt^* was amplified by PCR using “upstream” and “downstream” primer sets. The 3′ (bottom strand) primer of the “upstream” primer set and the 5′ (top strand) primer of the “downstream” primer set corresponded to the upstream and downstream halves of the GLS, respectively, and overlapped at a synthetic *Sal I* restriction site. The primers were designed to introduce a total of 12 single nucleotide mutations into the GLS (See Fig. 3), but targeted wobble positions and thus maintained the wild-type Grk protein sequence (Fig 3). Following PCR, the upstream (*Sap I – Sal I*) and downstream (*Sal I – Hind III*) PCR products were substituted for the *Sap I – Hind III* region of *grk^wt^* in a 3-way ligation reaction. The resulting *grkGLS^mut^* construct was cloned into the *Asp718 I – Xba I* sites of pCaSpeR4 as above.

### Wholemount *in situ* hybridization and immunostaining

Enzyme-linked *in situ* hybridization to wholemount ovaries was carried out according to Tautz and Pfeifle [45] with the modifications described in Cheung et al. [13]. Digoxigenin-labeled DNA probes were made by the random priming method [46]. The *K10* and *gurken* probes were as previously described [21], [25]. Photographs were taken with a Zeiss Axiophot and digitized by scanning with a Nikon LS-3510 film recorder or captured directly with a Leica DFC300 digital camera. Grk immunostains were carried out as previously described [38], [47] with a mouse anti-Grk monoclonal antibody [48] diluted at 1∶100 with PBS. Donkey anti-mouse secondary antibodies were purchased from Jackson labs and used at the manufacturer's recommended concentrations. Stained ovaries were mounted in 4% n-propyl gallate (Sigma) in 90% glycerol, 10% phosphate buffered saline. Images were collected on an Olympus 3L Spinning disc or a Zeiss Meta 510 laser scanning confocal microscope.
